# Top 5 Advances in the Last 50 Years: Malignant Gastric and Esophageal Surgery

**DOI:** 10.1002/wjs.70306

**Published:** 2026-04-11

**Authors:** Luís Santos Castro, Lorenzo Ferri

**Affiliations:** ^1^ Division of Thoracic and Upper Gastrointestinal Surgery Department of Surgery McGill University Montreal Quebec Canada

## 1 Introduction

We celebrate the 50th anniversary of the World Journal of Surgery and, with it, half a century of impactful research and communication. Publication and shared knowledge are the building blocks of modern medical and surgical practice. It is fitting that we publish this selection of great advances in esophageal and gastric cancer surgical treatment on the coincident 50th anniversary of the publication by KC McKeown of the description of the procedure that carries his name [1].

The following sections highlight what we considered to be the most relevant and practice‐changing advances in the field. This brief selection is not without its injustices as many important contributions had to be left out. As we write it, we pay homage to the collective memory and legacy of oncological surgery of the upper digestive tract.

### 1.1 Minimally Invasive Approaches for Esophageal and Gastric Cancer

The surgical management of both gastric and esophageal cancer has greatly evolved transitioning from high‐morbidity open procedures toward minimally invasive surgery (MIS) [2, 3]. Open resection was historically associated with significant postoperative complication and mortality rates, and encompassed a long recovery period for the survivors. The refinement of the surgical approach has been driven by the goal of reducing surgical trauma while maintaining oncological safety and efficacy [4].

Applying minimally invasive techniques to the resection of the esophagus and stomach has had champions from both the West and the East. James D. Luketich played a pivotal role in popularizing minimally invasive esophagectomy (MIE) in the West. In a landmark early series of 77 patients, Luketich and colleagues demonstrated that a combined thoracoscopic and laparoscopic approach was technically feasible and safe. This technique, which utilized four thoracoscopic ports and five abdominal ports, resulted in a median hospital stay of 7 days (compared to the 12 days stays seen with open surgery at the time) and no 30‐day operative mortality. Luketich's combined approach improved lymph node dissection and yield as well as thoracic esophagus mobilization [3]. By 2012, in a review of 1000+ patients treated, observed mortality rate was 1.4% establishing MIE as a procedure capable of lowering mortality in high‐volume centers [2]. A totally minimally invasive Ivor Lewis esophagectomy with an intrathoracic anastomosis was the preferred approach to reduce recurrent laryngeal nerve injuries and anastomotic leaks associated with cervical anastomoses [4]. The randomized TIME trial subsequently showed MIE resulted in significantly fewer pulmonary infections and better quality of life compared to open [5].

From the East, the pioneering work of Harushi Osugi at the Osaka City Medical College, Harushi Udagawa at Toronomon Hospital, and Yuko Kitagawa at Keio University, all demonstrated that the proven benefits of radical en‐bloc esophagectomy in terms of improved local and regional control, can be a achieved safely via a minimally invasive thoracoscopic approach [6, 7, 8].

Despite the success of MIE, the procedure is not without a steep learning curve estimated at around 100 cases to achieve proficiency in intrathoracic anastomosis. Future approaches to esophageal surgery revolve around robotic‐assisted MIE (RAMIE) to overcome the learning and technical limitations of standard MIE. The robotic platform offers an array of integrated tools such as 3D visualization and increased degrees of freedom, facilitating complex tasks like lymph node dissection in the upper mediastinum, near‐infrared fluorescence imaging for perfusion assessment, among other technologies. Furthermore, the integration of artificial intelligence and image recognition into robotic systems represents the next frontier, promising to further provide real‐time information and, ultimately, enhance surgeon performance and clinical outcomes [5].

Parallel to the MIS revolution, the surgery for gastric cancer shifted from open to laparoscopic gastrectomy. A 2016 Cochrane review noted that although laparoscopic gastrectomy showed no significant difference in short‐term mortality and resulted in shorter hospital stays, the quality of evidence regarding long‐term oncological outcomes was considered low [9]. However, subsequent large‐scale clinical trials have validated the oncological safety and efficacy of MIS in gastric cancer. The KLASS‐01 trial was pivotal in establishing laparoscopic distal gastrectomy as standard‐of‐care treatment for clinical stage I gastric cancer, demonstrating noninferior 5‐year OS rates while resulting in fewer complications. Following this observation, the role of MIS expanded to advanced gastric cancer with the KLASS‐02 trial showing that laparoscopic distal gastrectomy with D2 lymphadenectomy was oncologically sound, yielding comparable 3‐year disease‐free survival with significantly lower complication rates [10].

### 1.2 Endoscopic Management of Early Neoplastic Lesions of the Esophagus and Stomach

With the development of new endoscopic tools, the prevention and treatment of early esophageal and gastric cancer has moved significantly toward organ‐preserving endoscopic therapies. Within the right indications, this strategy yields minimal morbidity while maintaining oncological safety and clinical outcomes [11]. Described more than 2 decades ago, endoscopic radiofrequency ablation (RFA) proved effective in eliminating the metaplasic esophageal mucosa in Barrett's esophagus (BE), a known precursor of esophageal adenocarcinoma, therefore reducing its incidence [12, 13].

Considering resection of nodules or masses, snare‐based endoscopic mucosal resection (EMR) was the primary resection technique, albeit being largely limited to lesions smaller than 20 mm. Furthermore, piecemeal excision compromised the histological evaluation of the resection specimen and consequent pathological staging. The development of endoscopic submucosal dissection (ESD) in the early 2000s transformed the field by enabling en‐bloc excision of larger lesions with precise control over resection depth and mucosal margins [11].

In esophagus, treatment of dysplastic BE (low or high grade) and early adenocarcinoma (intramucosal node negative cancer) combines endoscopic resection techniques with RFA. Endoscopic eradication therapy is the standard‐of‐care for high‐grade dysplasia and intramucosal cancer due to the low risk of lymph node metastasis [14]. ESD is utilized for bulky lesions or those with suspected submucosal invasion, whereas EMR is employed for resection of small dysplastic nodules being safer and less costly than ESD. In the case of a residual BE segment, resection is typically followed by RFA. This strategy has proven effective in achieving complete eradication of intestinal metaplasia and subsequently reducing neoplastic progression [12, 13]. Esophageal ESD carries the risk of stricture formation, especially when the resection field is extensive. The management of this complication is amenable to subsequent endoscopic treatment, and the benefits of this minimally invasive organ‐preserving approach far outweigh its risks [15].

Early gastric cancer presents a similar indication for ESD, and this technique has become the preferred first‐line treatment. Surgery is reserved for cases in which ESD is deemed not feasible or specimen pathological criteria for endoscopic curative resection are not met [11].

### 1.3 Neoadjuvant Therapies

The therapeutic management of locally advanced resectable esophageal and gastric cancer has seen a significant shift in the last couple of decades from surgery alone to multimodal strategies. Meta‐analyses established that both neoadjuvant chemotherapy and neoadjuvant chemoradiotherapy (nCRT) improved survival compared to surgery alone, though the superiority of one modality over the other was initially unproven [16].

Since 2012, the CROSS trial set the standard for nCRT. It demonstrated that administering carboplatin and paclitaxel with concurrent radiotherapy (41.4 Gy) prior to surgery significantly improved overall survival (OS) for both squamous cell carcinoma and adenocarcinoma of the esophagus and esophagogastric junction (EGJ). Long‐term follow‐up confirmed this survival benefit [17].

Parallel to this, perioperative systemic chemotherapy regimens were developed. Published in 2019, the FLOT4 trial established perioperative FLOT (fluorouracil, leucovorin, oxaliplatin, and docetaxel) and resection as the standard‐of‐care for gastric and EGJ adenocarcinoma, demonstrating improved OS compared to the previously standard ECF/ECX regimens (MAGIC regimen) [18].

Recent phase 3 trials have sought to settle the debate between these optimized chemotherapy and chemoradiotherapy neoadjuvant approaches. The Neo‐AEGIS trial compared the CROSS regimen against perioperative chemotherapy (MAGIC or FLOT) for adenocarcinoma. Although nCRT achieved higher rates of pathological complete response and negative resection margins (R0), this did not translate into a survival benefit, leading investigators to conclude clinical equipoise between the approaches [19]. Another landmark trial, the TOPGEAR trial, showed that adding chemoradiotherapy in the preoperative setting to perioperative chemotherapy for gastric and EGJ cancer did not improve survival compared to chemotherapy alone [20].

However, recent data indicate a benefit of perioperative chemotherapy over nCRT for esophageal adenocarcinoma. The 2025 ESOPEC trial provided a direct comparison between perioperative FLOT and CROSS nCRT for this condition. The researchers found that FLOT significantly improved outcomes with a median OS of 66 months compared to 37 months in the nCRT arm [21]. Consequently, although nCRT remains a valid option, perioperative FLOT is now considered the superior treatment for locally advanced resectable esophageal adenocarcinoma.

### 1.4 Immunotherapy

The role of immunotherapy in esophagogastric cancer has evolved rapidly, moving from a salvage therapy in the setting of disease progression to first‐line metastatic treatment and, more recently, potentially curative‐intent strategies. Initially, immune checkpoint inhibitors were approved for chemotherapy‐refractory advanced disease based on trials such as ATTRACTION‐2 and KEYNOTE‐059. However, recent trials have integrated these agents into earlier lines of therapy. Biomarker testing, such as microsatellite instability (MSI) and programmed death ligand 1 (PD‐L1), is employed to select patients most likely to draw benefit from their use [22].

In the metastatic setting, immunotherapy combined with chemotherapy is now a standard first‐line approach. The CheckMate 649 trial demonstrated that adding nivolumab to chemotherapy significantly improved OS and progression‐free survival in patients with advanced gastric and EGJ adenocarcinoma. Similarly, the KEYNOTE‐590 trial established pembrolizumab plus chemotherapy as an alternative standard first‐line therapy particularly in patients with high PD‐L1 expression [22].

Immunotherapy has also made its way toward the management of locally advanced resectable disease. The landmark CheckMate 577 trial established adjuvant nivolumab as the standard‐of‐care for patients with resected esophageal or EGJ cancer who demonstrate residual disease on resection specimen following nCRT. In this population, adjuvant nivolumab doubled the median disease‐free survival compared to placebo (22.4 vs. 11.0 months) [23]. More recently, the phase 3 MATTERHORN trial evaluated the effect of perioperative immunotherapy for resectable gastric and GEJ adenocarcinoma. Adding durvalumab to standard FLOT chemotherapy significantly improved 2‐year event‐free survival (67.4% vs. 58.5%) and increased pathological complete response rates (19.2% vs. 7.2%) compared to chemotherapy alone [24].

Finally, specific strategies emerged for deficient mismatch repair, i.e., MSI‐high tumors. The 2022 NEONIPIGA phase II study showed that neoadjuvant nivolumab plus ipilimumab followed by adjuvant nivolumab resulted in a pathological complete response rate of 58.6% [25]. These promising results open the door to organ‐preservation strategies for those patients who achieve a complete clinical response.

### 1.5 Conceptualization of Oligometastatic Disease

The concept of oligometastatic disease (OMD) in esophageal cancer has evolved from a theoretical biological spectrum to a clinically distinct entity with standardized management guidelines. The term was originally coined in 1995 by Hellman and Weichselbaum to describe an intermediate state between localized and widespread polymetastatic disease where metastases are limited in number and aggressiveness. This “spectrum theory” postulates that metastatic capacity is acquired incrementally, and local control of limited metastases could yield survival benefits or even cures, challenging the traditional dogma that metastatic disease warrants only palliative‐intent systemic therapy [26, 27].

For a long time, the implementation of this concept was hindered by the lack of a consistent definition for OMD in esophagogastric cancer. This led to heterogeneous study populations and difficulties comparing outcomes [28, 29]. To address this variation, the OligoMetastatic Esophagogastric Cancer (OMEC) project was established to create a uniform consensus. Based on recent European guidelines, OMD is now strictly defined as disease involving one organ with three or fewer metastases or one involved extra‐regional lymph node station. This definition explicitly excludes polymetastatic presentations, such as peritoneal carcinomatosis, which entail distinct treatment strategies. This standardization aims to improve concept definition and, ultimately, patient selection for future randomized trials [26].

The therapeutic approach has changed from a one‐size‐fits‐all palliative strategy to a multimodal one. Although recurrent or metastatic esophageal cancer was historically deemed incurable, evidence now indicates that aggressive local treatments, such as metastasectomy or stereotactic body radiotherapy (SBRT), can significantly improve survival [30].

Current guidelines emphasize a tailored approach based on patient and disease characteristics. For patients with synchronous OMD (or metachronous disease with a short disease‐free interval—less than 2 years), the recommended approach is systemic therapy followed by restaging and consideration for local consolidative treatment [26].

## Author Contributions

**Luis Santos Castro:** conceptualization, writing – original draft. **Lorenzo Ferri:** conceptualization, supervision, writing – review and editing.

## Conflicts of Interest

The authors declare no conflicts of interest.

## Data Availability

The data that support the findings of this study are available on request from the corresponding author. The data are not publicly available due to privacy or ethical restrictions.
